# A simple and affordable kinetic assay of nucleic acids with SYBR Gold gel staining

**DOI:** 10.1371/journal.pone.0229527

**Published:** 2020-03-03

**Authors:** Danielle Guillen, Mika Schievelbein, Kushkumar Patel, Davis Jose, Jonathan Ouellet

**Affiliations:** Department of Chemistry and Physics, Monmouth University, West Long Branch, New Jersey, United States of America; Sichuan University, CHINA

## Abstract

Labeling substrates or products are paramount in determining enzymatic kinetic parameters. Several options are available; many laboratories use either radioactive or fluorescent labeling because of their high sensitivity. However, those methods have their own drawbacks such as half-life decay, expensive and hazardous. Here, we propose a novel, simple, economical and fast alternative to substrate labeling for studying the kinetics of nucleic acids: post-migration gel staining with SYBR Gold. Cleavage rates similar to the ones reported in the literature for the I-R3 DNA-cleaving DNA enzyme in the presence of zinc chloride are an indication of the quality of the new method. Moreover, the activity of the hammerhead ribozyme was also monitored by our method to illustrate its versatility. This labeling-free method has several advantages such as its ease of use as well as cost effective and versatility with both non-structured and structured RNAs or DNAs.

## Introduction

The study of traditional biochemistry through kinetics is centered about the understanding of enzymes as catalysts with their kinetic parameters k_cat_ and K_M_ [1]. Of the generally known enzymatic reactions in an organism, fewer than 10% of those have had their kinetic parameters determined by *in vitro* enzyme kinetics [1], hence there is a need to increase the biochemical toolbox to monitor kinetics.

Many techniques are available to establish the kinetic parameters, which is often done for structure-function analysis. Agarose gels or polyacrylamide gel electrophoresis (PAGE) in native/denaturing conditions with either SDS or urea are commonly used in kinetic studies of nucleic acids and proteins [2,3]. In most cases, substrate-labeled nucleic acids that are separated in gels are then revealed by autoradiography films, or by biomolecular imager screens. Common radioisotopes are 32-P for its high intensity and sensitivity, while 35-S was preferred for manual dideoxy DNA sequencing. The advantage of using 32-P in DNA or RNA is that it does not change its chemical nature because it has the same size and charge as any other phosphorus in the nucleic acid backbone. Radiolabeled substrate kinetic assays are considered discontinuous: small aliquots are removed before being separated by gel electrophoresis. Although the detection of labeled molecules is very sensitive, the quality of the data depends on the number of discontinuous datapoints collected. An alternative to radioactive labeling is fluorescence labeling, followed by migrating the aliquots on gels, and recording with a biomolecular imager [4]. In contrast with conventional radioisotope experiments, reactions could also be recorded continuously by stop-flow spectrofluorometry. While radioactivity gel kinetics provides discontinuous and fluorescence spectroscopy can provide continuous data sampling, recent advances in deep sequencing also have allowed large scale mutant profiling and simultaneous rate constant measurements for more than 4000 reactions at multiple time points [5]

The recording of kinetics for nucleic acids generally involves fluorescence spectrophotometry or gel electrophoresis with labeled substrates [6]. Continuous data acquisition in fluorescence spectroscopy provides powerful precision due to its number of datapoints. However, the bulkiness and charges of the fluorophores are of critical concern in nucleic acid structure-function analysis, as it has been shown to interfere with kinetic activity of ribozymes [7]. Further, the initial investment for acquiring the spectrofluorometer and the high-quality quartz cuvettes, as well as the continuous purchase of fluorescently-labeled nucleic acids may be a deterrent. The major advantage of using fluorophores over conventional 32-P radiolabeled substrate nucleic acid is its stability over time whereas 32-P radiolabeled substrates inherently have a half-life decay of 14.3 days. Regulations, permits, (de)-contamination, storage of sources, waste management and usage safety are major concerns in using radioisotopes. Similarly for fluorescence measurements, the cost of a PhosphorImager (or its equivalent) and its associated screens are another major investment. More importantly, both methods require careful purification of the labeled substrates which considerably increase sample preparation time.

Either radioactivity or fluorescence studies routinely require labeling the substrate followed by its purifications, precipitations, as well as occasional Sephadex G50 desalting columns or preparative HLPC purification, which are inherently time-consuming. Development of a nucleic acid visualization method without prior substrate labeling would lead to simpler and more convenient kinetic studies, without involving health-hazardous or bulky molecules.

As conventional gel kinetics would usually have its substrate covalently labeled with a radioisotope or a fluorophore, very few examples of extrinsic fluorescent probes have been successfully published for nucleic acids enzymology. Examples of extrinsic fluorescent probes have been reported to monitor the 10–23, as well as the 8–17 DNAzyme kinetics, by spectrofluorometry using ethidium bromide [8] or PicoGreen [9]. The kinetic monitoring with either fluorophore is efficient because they intercalate into the long stable duplex stems of the DNAzyme, without affecting the structure of the core. However, both fluorophores failed to monitor the kinetic progress of highly structured complexes such as the hammerhead ribozyme.

According to the manufacturer, the asymmetrical cyanine dye SYBR Gold Nucleic Acid Gel Stain is 25–100 times more sensitive than ethidium bromide, with a detection minimum of 25 pg DNA. Binding of SYBR Gold to double-stranded, or single-stranded, DNA or RNA increases its fluorescence signal by more than 1000-fold. Multiple excitation maxima permit fluorescence in the UV-range as well as in the blue range. SYBR Gold likely binds the charged phosphate backbone of nucleic acids and remarkably, can stain DNA and RNA in denaturing urea, formaldehyde, or glyoxal gels [10].

Among the wide array of nucleic acid models available to perform structure-function studies, the self-cleaving ribozymes are commonly used for conventional radioactive gel-kinetics[11]. With the aim of selecting a nucleic acid model that is stable and affordable, a DNA system is more advantageous than an RNA system. Although the DNAzymes such as the 10–23 and the 8–17 are mostly made of DNA, their substrates require at least a single RNA base at the cleavage site, making the mixed DNA-RNA polymer less than optimal. Considering its stability, ease of access and low-cost, a DNA enzyme and substrate would constitute the simpler nucleic acid model to allow the troubleshooting of our alternative quantification method.

**Fig 1:**
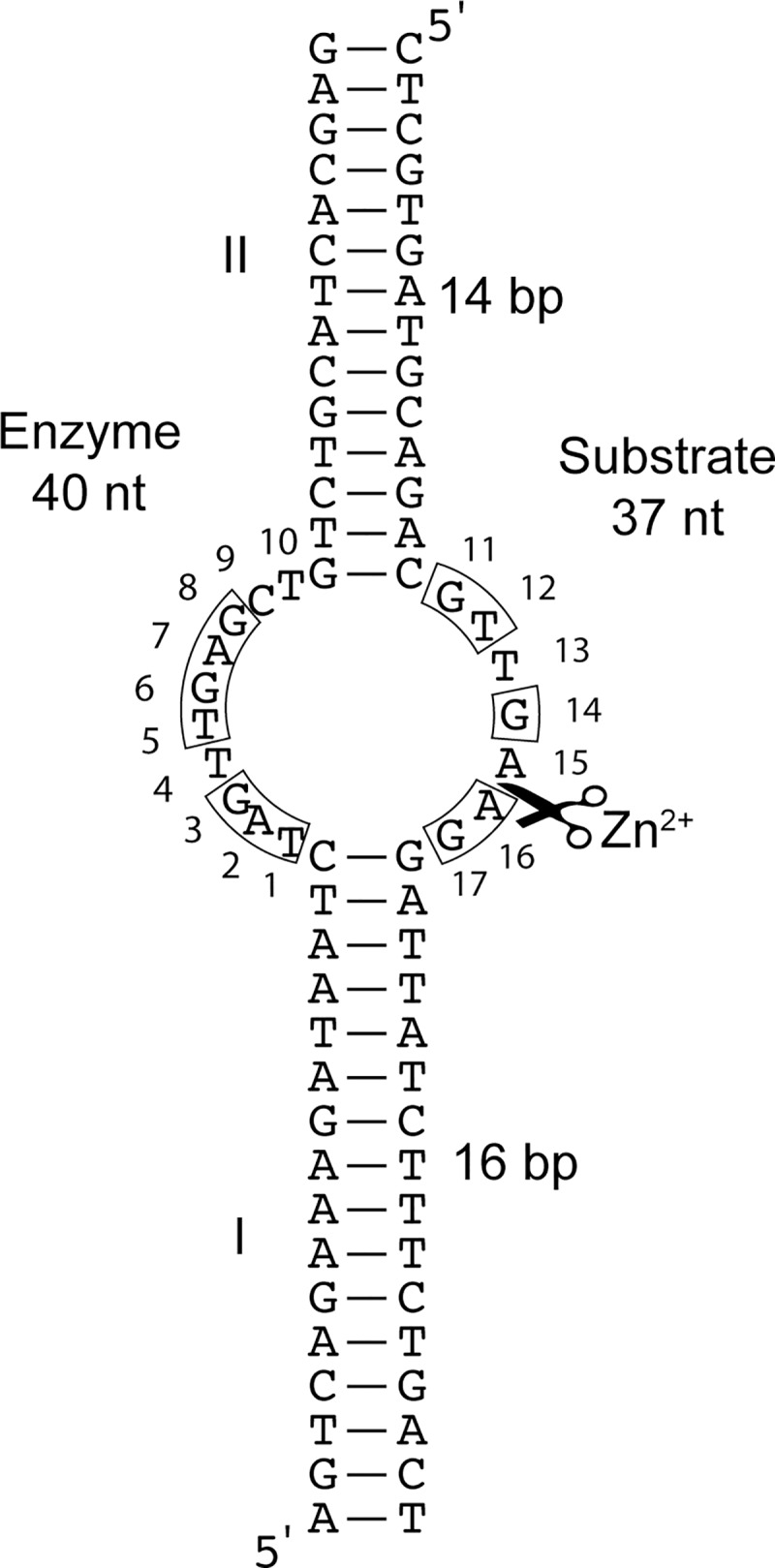
2D structure of the D-Zyme DNAs. The D-Zyme DNAs, the DNA enzyme (E) of 40 nt-long, annealed to the 37 nt-long DNA substrate (S). The two strands are complementary base paired on the two terminal stems I and II, and are not paired in the catalytic core. In the presence of zinc chloride, the substrate strand is cleaved between the two adenines at positions A15 and A16 (illustrated with scissors), creating two products: the 5’-end product P1 of 19 and the 3’-end product P2 of 18 nucleotide-long. The boxed nucleotides indicate the conserved core of the D-Zyme.

Such catalytic DNA enzyme, initially named I-R3, self-cleaves and was recently discovered by systematic evolution of ligands by exponential enrichment (SELEX) [12]. Particularly, this DNA-only catalytic model exhibits cleavage activity strictly in presence of zinc ions. The self-cleaving DNA has been engineered as a bi-molecular enzyme (that we named D-Zyme to differentiate it from the cis-acting version) with substrate base pairing *via* two pairing DNA stems I and II, interrupted by a central asymmetric bulge (Fig 1). Such trans-acting DNA enzyme (E) and its substrate (S) becomes the D-Zyme once annealed. The SELEX variants identified a core of 17 nucleotides, where 15 of them seem to be conserved, including a cleavage site between A15 and A16. Deep sequencing mutational profiling for more than 4000 variants revealed catalytically essential nucleotides as well as tolerated mutations within the core [5]. This D-Zyme was further reselected to improve its multiple turnover activity as well as to assess its temperature dependence [13]. The D-Zyme kinetic constants have been determined by conventional radioactive gel kinetics as well as deep sequencing profiling [5, 12]. A DNA-only system such as this D-Zyme is a simple and stable DNA model that is ideal for rapid and inexpensive troubleshooting of the alternative quantification. With the aim of establishing a method that is versatile, we also demonstrated its use with the all-RNA hammerhead ribozyme.

Herein, we are reporting an alternative to a conventional gel-kinetic method to quantify nucleic acids in gels after staining with the extrinsic fluorophore SYBR Gold. Such a post-migration gel staining method for visualization and quantification of nucleic acids does not require prior substrate labeling as well as providing a fast, easy and affordable alternative to radioactivity discontinuous data sampling. Whereas the alternative method intent was not to offer new data or finding into the field, the validity of the method is demonstrated by the cleavage constant rates of the D-Zyme being similar to other research groups using radioactivity and its versatility is confirmed with a hammerhead ribozyme kinetic monitoring.

## Material and methods

All chemicals for solutions and buffers were purchased from Sigma-Aldrich (SL, MO), SYBR Gold was purchased from Molecular Probes by Life Technologies (Eugene, Oregon) and the transcription was performed according to the manufacturer with the High Yield RNA Polymerase kit from New England Biolabs (Ipswich, MA). The oligonucleotides were purchased from Integrated DNA Technologies (Coralville, Iowa) and are listed in Table 1. The software ImageJ (version 1.52K, NIH) was used to measure the band densitometry.

**Table 1:** Name and sequence of DNA oligonucleotides. The nucleotide “X” represents the fluorescent adenine analogue 2-AP. The T7 promoter sequence to produce RNA is in italics uppercase, while the DNAs encoding for the ribozyme RNAs are in lowercase. The nucleotides encoding the 1/2 junction of catalytic hammerhead core are highlighted in bold.

Oligo name	Sequences 5ʹ to 3ʹ	
E (40 nt)	AGTCAGAAAGATAATCTAGTTGAGCTGTCTGCATCACGAG	
S (37 nt)	CTCGTGATGCAGACGTTGAAGGATTATCTTTCTGACT	
S 2AP (37 nt)	CTCGTGATGCAGACGTTG**X**AGGATTATCTTTCTGACT
S -4 (33 nt)	CGTGATGCAGACGTTGAAGGATTATCTTTCTGA	
HH RzB F	*TAATACGACTCACTATA*gggacttaagccca**ctgatga**gtggctgggatgcgacgaaacgcc	
HH RzB R	cgggcgtttcgtcgcatcccagccac**tcatcag**tgggcttaagtccc*TATAGTGAGTCGTATTA*	
HH Sb F	*TAATACGACTCACTATA*gggcgtctgggcagtccc	
HH Sb R	gggactgcccagacgccc*TATAGTGAGTCGTATTA*	

### Slow annealing of DNA in buffered solution

The homogeneity of the structural intermediates was ensured by slow cooling to the reaction temperature from a high denaturation temperature [6]. The reaction buffer allows the DNA to pre-fold without DNA cleavage. The annealing of the bimolecular DNA strands (10:1, enzyme:substrate) was performed in a reaction buffer (50 mM HEPES in 100 mM NaCl, pH adjusted to 7.05) by heating for 1 min at 85°C followed by slow cooling in an aluminium block for several hours (minimum of 5 hours to overnight).

## D-Zyme and hammerhead ribozyme kinetic measurements

The cleavage assay, at 25°C, was initiated by adding a solution to 20 mM ZnCl_2,_ 50 mM HEPES in 100 mM NaCl with pH adjusted to 7.05, to the annealed DNA in reaction buffer, for a final concentration of 20mM ZnCl_2_ and several different zinc concentrations were tested. At specific time increments, aliquots were quenched in 8 μL of chilled STOP solution (98% Formamide, 10 mM EDTA, 0.025% bromophenol blue, 0.025% xylene cyanol). DNA was resolved, according to size, on a 14x20 cm glass plate set, 20% PAGE 8M urea for 120 minutes at 20W in TBE buffer.

The hammerhead ribozyme and substrate [7] for cleavage activity experiments was synthesized by transcription using T7 RNA polymerase [14] from double-stranded DNA templates. Templates for transcription of ribozymes were made by slow annealing of DNA oligonucleotides in 10 mM Tris pH 8.3, 50 mM KCl and 1.5 mM MgCl_2_. RNAs were purified on urea PAGE and was recovered by passive elution. Eluted RNAs were then recovered by ethanol precipitation and dissolved in water. The RNAs were refolded in a 50 mM Tris solution at pH 7.0 with a snap cooling, which was accomplished by heating 20 pmols of hammerhead substrate with 200 pmols hammerhead ribozyme to 80°C for 1 minutes followed by 3 minutes on ice. After 10 minutes pre-incubation at 37°C the cleavage reaction was initiated by adding 10 mM MgCl_2_ and aliquots were quenched in chilled STOP solution at various time intervals before migration on 8 M Urea PAGE for the cleavage analysis and curve-fitting.

### Gel staining and densitometry

SYBR Gold stained the gel for 30 minutes before UV transillumination at 302 nm and tiff pictures acquired *via* an Enduro Gel Dock system. Open source software, ImageJ (version 1.52K, NIH) was then used to measures the band density for each DNA in order to calculate percent cleavage. The areas under the curves were exported to a spreadsheet to calculate cleavage percentages. This process was repeated for each lane.

### Cleavage analysis

Values for percent cleavage of DNA were determined by calculating the ratio of cleaved DNAs over the total amount of substrate and product DNAs (Eq 1).

$$\%=\frac{P_{1}+P_{2}}{P_{1}+P_{2}+S}$$ (1)

Where P_1_ and P_2_ are the product intensities and S is the uncleaved substrate intensity.

Nonlinear regression of the data was fitted to a single exponential in Minitab (version 18.1, Minitab, Inc.), according to Eq 2, to determine cleavage rates, k_obs._

$$y=-A\cdot e^{\left(r\cdot x\right)}+B$$ (2)

Where *A* represents amplitude, *r* represents rate, and *B* represents the extent of cleavage.

### D-Zyme spectrofluorometry

Steady-state fluorescence spectra of 2-AP-labeled D-Zyme substrate annealed to a ten-fold excess of D-Zyme enzyme in 50 mM NaCl and HEPES pH 7.05. with and without zinc chloride were recorded on FS5 Spectrofluorometer from Edinburgh Instruments Ltd (UK) at 1.5 nm bandwidth and 0.5 seconds dwell time.

The Fluorescence kinetics were recorded by mimicking stopped-flow fluorometry: the instrument lid was opened to add an equal sample volume of zinc chloride, while the data acquisition was still on-going. A 150 μL quartz fluorescence cuvette (Starna Cells, California, USA), contained a volume of 50 μL with 1 μM of 2-aminopurine (2-AP) substrate and 10 μM enzyme in 50 mM NaCl and HEPES pH 7.05. After recording for 5 minutes in the temperature-controlled fluorometer sample holder at 25°C, addition of the zinc chloride initiated the reaction. The instrument recorded a datapoint every 0.5 second for λ_ex_ at 305 nm and λ_em_ at 340 nm for 25 minutes with a bandwidth of 2.0 nm.

## Results

### SYBR Gold gel staining, UV transillumination and densitometry

**Fig 2:**
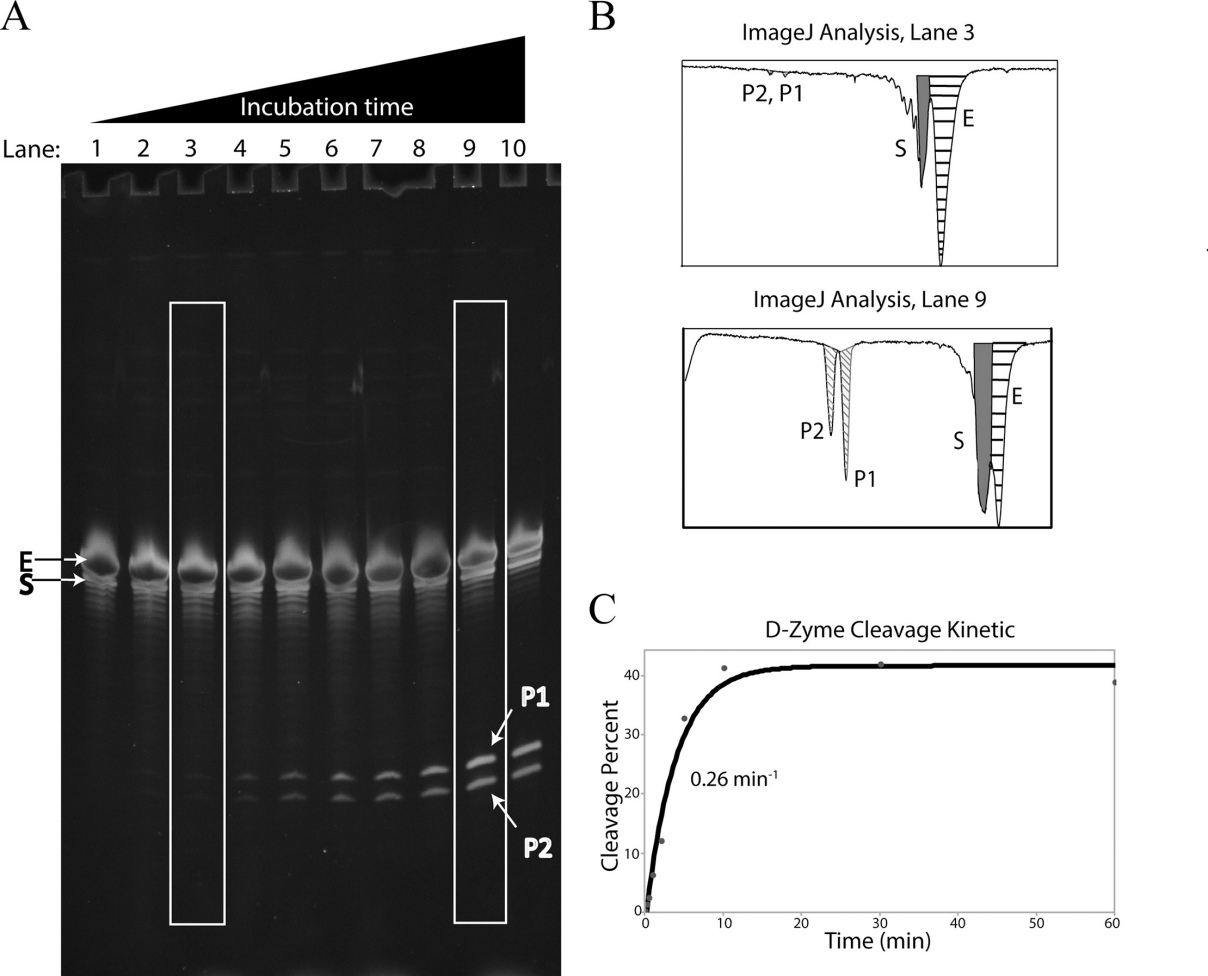
**A**. Kinetics of the D-Zyme, viewed on a 20% PAGE. Incubation time increases in each well from left to right, with 0 sec in lane #1, 20 sec in lane #2, 40 sec in lane #3, 60 sec in lane #4, 2 min in lane #5, 3 min in lane #6, 5 min in lane #7, 10 min in lane #8, 30 min in lane #9 and 60 min in lane #10. The enzyme strand (E) is shown at 40 nt length followed by the substrate strand (S) at 37 nt, and the two products at (P2) 18 and (P1) 19 nt. Selected lanes highlight the significant difference in band intensity between two time points, correlating to an increased amount of product as the reaction progressed. **B**. ImageJ graphs that corresponds to the lanes highlighted. As time increases, total cleavage increases. Diagonal hashed segments represent the products (P2 and P1), dark gray represents the substrate (S), and horizontally striped represents the enzyme (E). Although the enzyme is highlighted into the figure, it should be noted its density is not used in calculating percent cleavage. **C**. Representative single exponential curve-fitting using Minitab of densitometry analysis for D-Zyme kinetics.

We determined that a 20% PAGE was optimal for clearly separating the lengths of the enzyme (E), substrate (S), and both products P_1_ and P_2_ (40, 37, 19, and 18 nucleotides respectively, Fig 2A). In such gels, one might see the “S” band intensity is not increasing as the reaction proceed. In fact, visual examination of product accumulation is indistinguishable and only image analysis software counting pixels was effective in determining cleavage rates.

Critically important, the quantity of product observed must be detectable with the staining dye, as it will dictate the amount of substrate needed, which in turn, defines the amount of enzyme required for single-turnover conditions. The detection and quantification of the minimal amount of 18-nucleotide single-stranded DNA (ssDNA) that cannot only be detected, but reliably quantified, was determined for various dyes and our measurements showed that SYBR Gold can efficiently detect and quantify as low as 1 picomole of a short DNA product, whereas the detection limit of approximately 0.2 pmol, provided by the manufacturer, cannot be precisely quantified [10].

To determine the quantity of DNA loaded into each well, we estimated that a low cleavage activity from an initial 30 pmols of substrate should provide at least 1 pmol of product. A kinetic reaction with 10 aliquots calls for a reaction mixture of 3300 pmols of DNAs for the 10:1 single-turnover ratio.

Apart from the non-labeled substrate and the large quantity of DNAs required, the kinetic cleavage reaction for this new quantification method is done in a very standard way, with: i) slow-cooling annealing of the enzyme and substrate DNAs in a buffered solution to permit stable folding followed by incubation at 25°C; ii) initiation of the reaction by adding zinc chloride to a final concentration of 20 mM; iii) at various times, aliquots were taken out from the reaction mixture and quenched in chilled formamide; iv) the aliquots are separated on 8 M Urea 20% PAGE.

After the DNAs were PAGE-separated according to sizes, the gel was placed in a glass square dish containing 7 μL SYBR Gold in 70 mL TBE solution. An orbital shaker distributed SYBR Gold into the gel, for 30 minutes at room temperature while being kept in the dark. According to the manufacturer, SYBR Gold does not require prior fixing nor de-staining. The gel was then placed directly onto the UV transilluminator at 302 nm, and tiff pictures (Fig 2) were acquired *via* an Enduro Gel Dock system.

Using this alternative radioactivity-free discontinuous kinetic method, the D-Zyme yielded an average cleavage rate of 0.57 ± 0.15 min^-1^ at 25°C. Table 2 summarize the four independent kinetics with less than ± 0.11 curve fitting error.

**Table 2:** *Trans*-acting cleavage activity of D-Zyme at 25°C, in 20 mM ZnCl_2_ at pH 7.05, with a 10:1 ratio of enzyme:substrate, quantified by post-migration SYBR Gold staining, analyzed by ImageJ and curve-fitted to a non-linear regression single-exponential function in MiniTab, as shown in Fig 2.

Independent Trial #	Rate (min^-1^)	Curve-fitting error
1	0.48	± 0.11
2	0.59	± 0.10
3	0.76	± 0.10
4	0.43	± 0.09
Avg. ± Std. Dev.	0.57 ± 0.15	

## 2-AP at the cleavage site

Previous work showed that a different nucleotide, other than deoxyadenine, located before the cleavage site at dA15 lowers the activity [12]. For instance, the substrate DNA dA15dC or dA15dG causes the catalytic rate to decrease from 1.0 min^-1^ to 0.067 min^-1^ or 0.38 min^-1^ respectively. A common chemogenetic base substitution in nucleic acids kinetic and structural studies is with deoxy 2-amino purine (2-AP). Such base substitution has structural similitudes to bode adenine and guanine, and more importantly, 2-AP fluoresce. A deoxyadenosine substitution for 2-AP at the cleavage site vicinity can therefore be for standard discontinuous kinetics as well as continuous fluorescence spectroscopy to provide a comparison between both methods. The regular dA15 was substituted with the fluorescent base 2-AP. Such chemogenetic modification caused an observed cleavage that was reduced from 0.57 min^-1^ to 0.21 min^-1^ (Table 3).

**Table 3:** *Trans*-acting cleavage activity of DNA Enzyme (E) with 2-AP substituted nucleotide at 25°C, in 20 mM ZnCl_2_ at pH 7.05, with a 10:1 ratio of enzyme:substrate, quantified by post-migration SYBR Gold staining, analyzed by ImageJ and curve-fitted to a non-linear regression single-exponential function in MiniTab.

Independent Trial #	Rate (min^-1^)	Curve-fitting error
1	0.26	± 0.04
2	0.15	± 0.03
3	0.21	± 0.03
Avg. ± Std. Dev.	0.21 ± 0.06	

**Fig 3:**
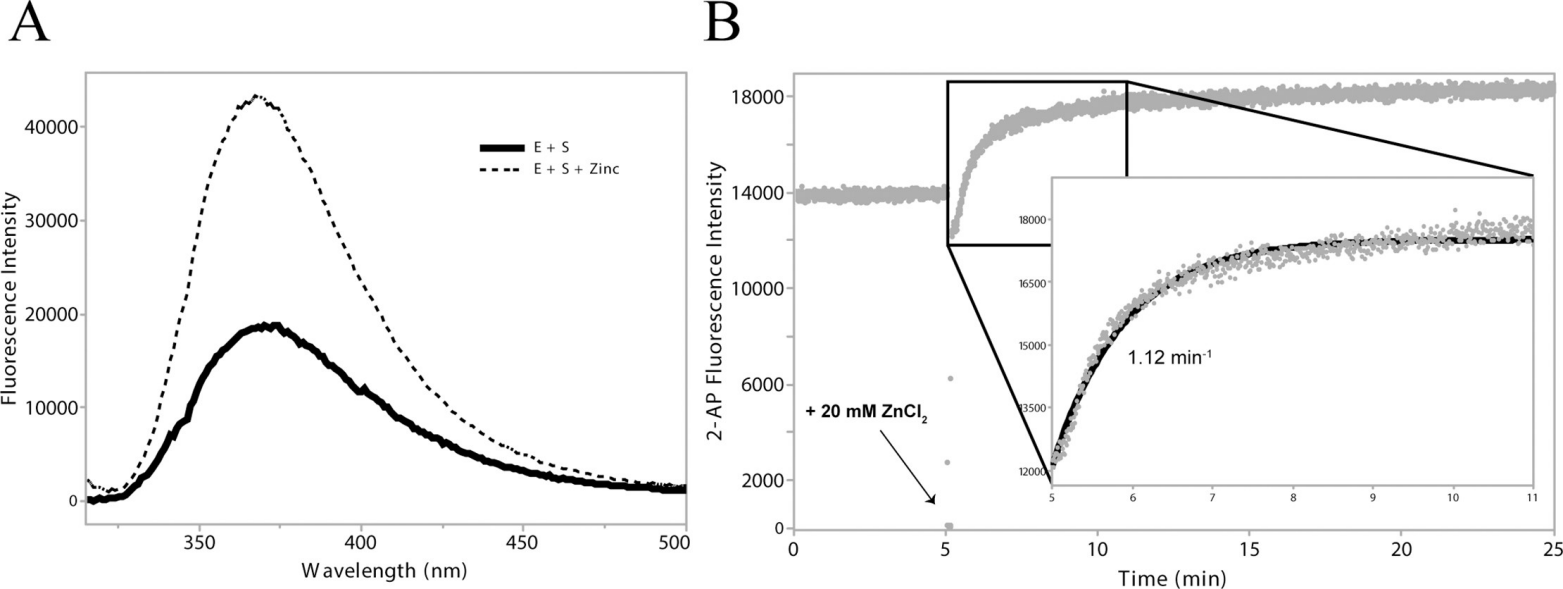
**A**. Steady-State fluorescence quenching of S 2AP. **A**. The DNA Enzyme annealed to the full-length 2-AP substrate, where the 2-AP is fluorescence in quenched (solid curve). Once the DNA enzyme has cut the 2-AP substrate after zinc chloride addition, the product has a higher fluorescence intensity (dashed curve). **B.** DNA enzyme and S 2AP kinetics, measured via fluorescence. After 5 minutes equilibration, the instrument lid is opened while the acquisition is still active, shown by fluorescence intensity drop to zero. The rapid addition of zinc chloride is indicated by an arrow. The closing of the lid prevents the acquisition of the very early datapoints of the kinetic. The single-exponential curve fitting still allows measurement of the activity rate, as seen in the inset.

In addition to discontinuous gel kinetics, the first D-Zyme steady-state fluorescence, as well as the continuous cleavage kinetics using a spectrofluorometer with the 2-AP at the cleavage site, were measured. As a full-length substrate, the 2-AP is stacked between G14 and A16 and has a low fluorescence intensity. After addition of zinc chloride, the cleavage of the DNA backbone between 2-AP and A16 disrupts the 2-AP stacking and the fluorescence intensity is drastically increased (Fig 3A). In single-turnover conditions, the substrate cleavage plateaued after 3 minutes. The instrument recorded a datapoint every 0.5 second, which lead to a single-exponential fit rate of 1.65 min^-1^ (Table 4, and S2 Fig).

**Table 4:** *Trans*-acting cleavage activity of DNA Enzyme (E) with 2-AP substituted nucleotide at 25°C, in 20 mM ZnCl_2_ at pH 7.05, with a 10:1 ratio of enzyme:substrate, monitored by kinetic spectrofluorometric measurement and curve-fitted to a non-linear regression single-exponential function in MiniTab.

Independent Trial #	Rate (min^-1^)	Curve-fitting error
1	1.12	± 0.02
2	1.84	± 0.03
3	1.97	± 0.04
4	1.66	± 0.04
Avg. ± Std. Dev.	1.65 ± 0.37	

### Zinc chloride dependence

**Fig 4:**
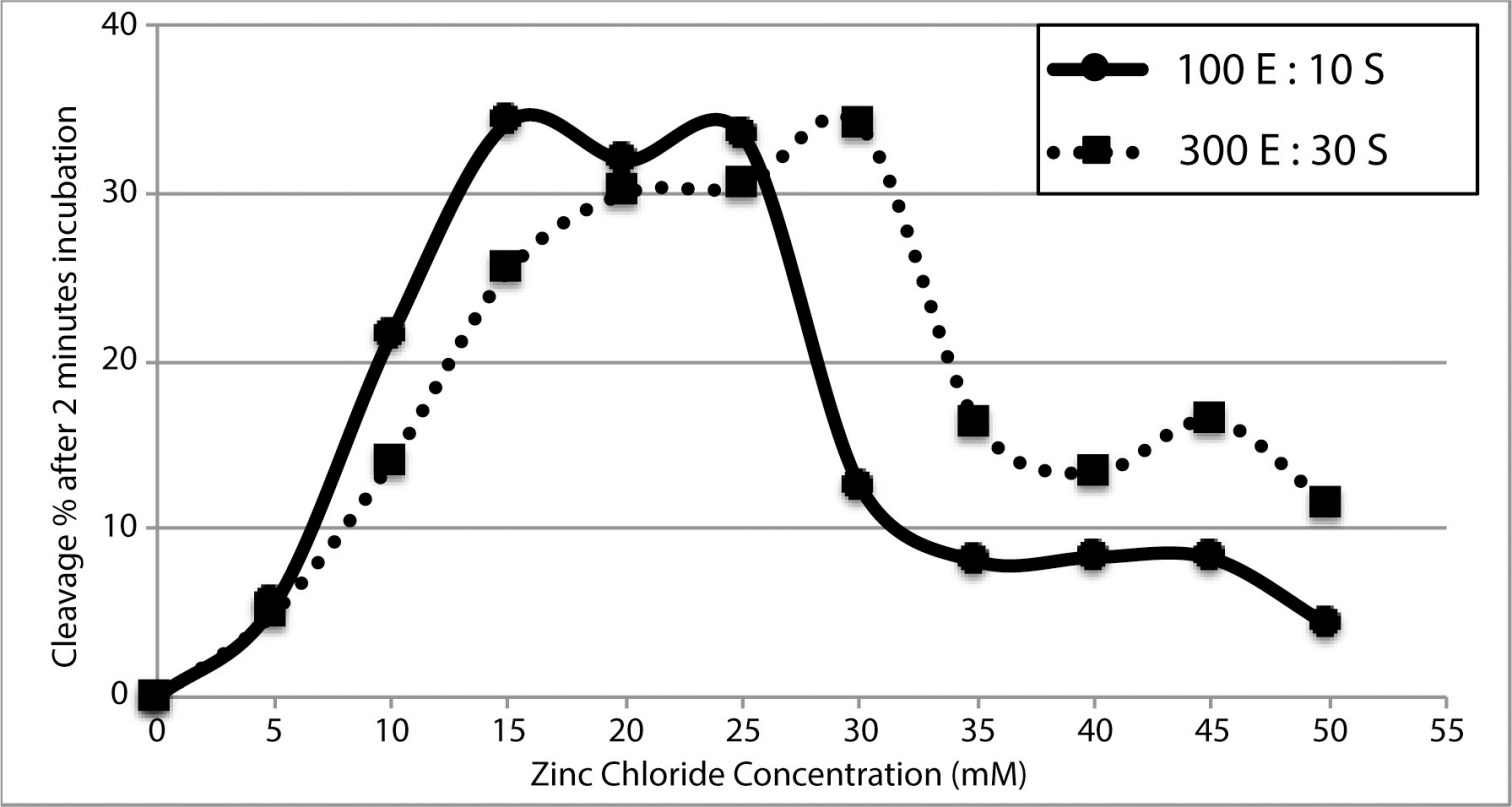
Cleavage percentages after 2 minutes incubation for an increasing concentration of zinc chloride. The dashed-curve with square datapoints indicates the D-Zyme activity when 330 pmols of DNA is in a single kinetic reaction while the solid-curve with circle datapoints has less DNA with 110 pmols. The final volumes of all reactions were maintained constant.

Prior work [12] showed that the optimal zinc concentration for 10 pmols of enzyme in the presence of 0.2 pmol radiolabeled substrate was between 1–2 mM, while 5–10 mM showed limited to no cleavage. After experimentation with various zinc and DNA concentrations, it was determined that 20 mM zinc was optimal for our experimental conditions while the 1–5 mM zinc concentrations were very ineffective (Fig 4). To evaluate the discrepancies between those zinc concentration dependence, we performed the kinetics with various DNA concentrations over a range of zinc concentrations. The major difference between the current and prior work is the total quantity of DNA used (3300 pmols and 10.2 pmols respectively), independently of the magnesium or sodium ion concentration [12].

To measure the effect of DNA concentration relative to the optimal zinc chloride concentration required for cleavage activity, the % cleavage after 2 minutes incubation at 25°C in 50 mM NaCl and HEPES pH 7.05 was measured for every 5 mM of zinc chloride from 0 to 50 mM. One series was made with 300 pmol of D-Zyme enzyme and 30 pmol Substrate while another one at 100:10, where the final volume for each reaction was identical. At the lower DNA concentration, the D-Zyme attains its maximum activity at 15 mM ZnCl_2_ while the concentrated D-Zyme requires 30 mM ZnCl_2_ to plateau (Fig 4). Our current hypothesis relies on the molecular crowding effect of highly concentrated samples. More investigations are underway to explore this topic.

### Hammerhead ribozyme cleavage assay

**Fig 5:**
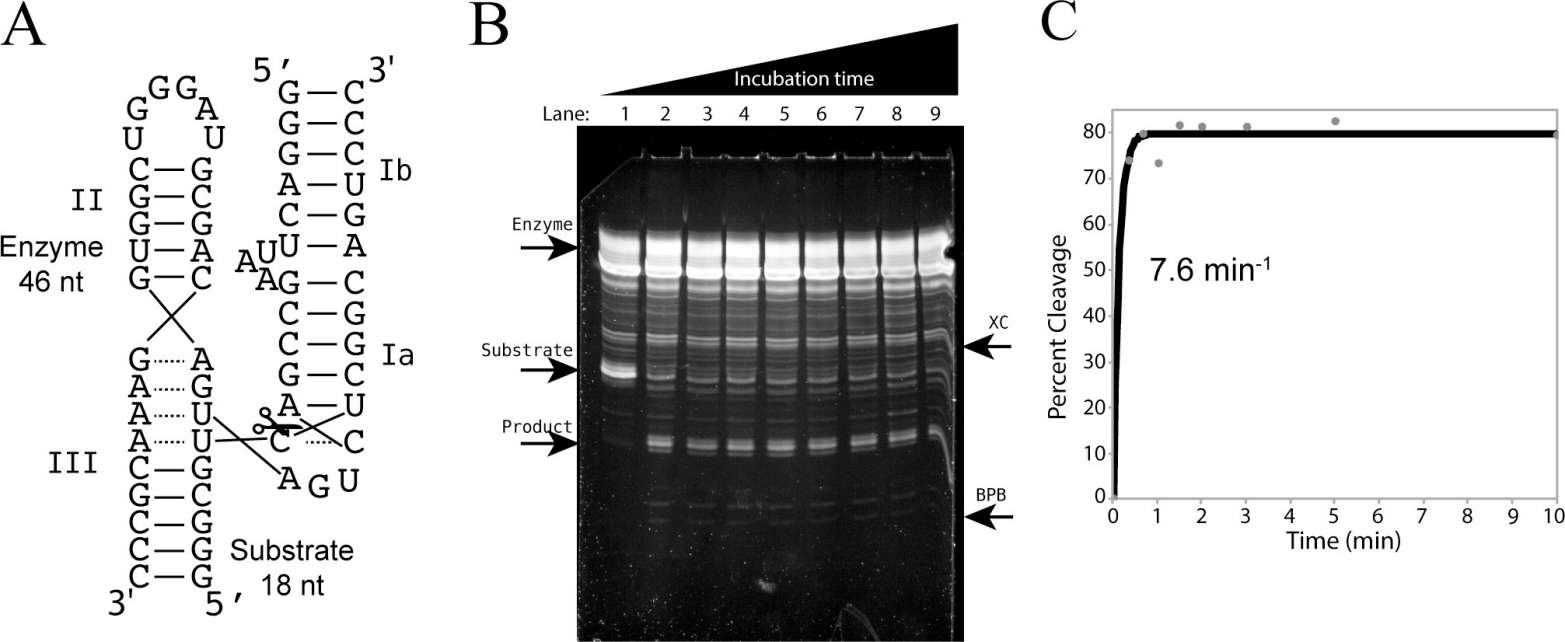
**A** 2D structure of the RzB hammerhead ribozyme, annealed to its cognate 18 nt-long substrate. In the presence of magnesium chloride, the substrate strand is cleaved into two products 7 and 11 nucleotides in length, illustrated with scissors. **B** 20% (19:1) polyacrylamide 8M urea gel separation of the kinetics of the RzB hammerhead ribozyme, substrate and products. The position of the xylene cyanol and the bromophenol blue have been marked with a UV-Shadowing picture prior to SYBR Gold staining. The stain identifies the substrate and the product as well as the ribozyme degradation products (which would usually not be seen in a radioactive experiment). **C.** Single exponential curve-fitting using Minitab of densitometry analysis for the *trans* hammerhead RzB kinetics. At 10 mM magnesium chloride, this RzB showed greater than 70% cleavage after 20 seconds, providing an estimated and inaccurate fast rate.

Previous spectrofluorometric kinetic measurements of DNAzymes with extrinsic dyes worked because of the relatively simple structural organization of long DNA stems [7,8]. However, the cleavage kinetics catalyzed by the structured hammerhead ribozymes with such extrinsic dyes could not be monitored. Kinetics using our new quantification method of post-migration gel staining with SYBR GOLD confirmed the fast-cleavage rate of the *trans* RzB hammerhead ribozyme[13], resulting in a cleavage rate of 7.6 min^-1^ (Fig 5). Such fast observed cleavage rate for the hammerhead ribozyme confirms the validity of this alternative kinetic method to measure kinetic rates of complex ribozymes.

## Discussion

### Extrinsic dyes for nucleic acid kinetics

In enzymatic kinetic studies, identifying the substrate or product is paramount for determining the kinetic parameters. Traditionally, such molecules have been labeled with radioisotopes or fluorophores, each with its *pros* and *cons*. Common concerns for radioisotopes are their health hazards, stringent regulations, and half-life decay, while the fluorophores are bulky and can modify the charge of the analyte being studied and thus interfere with structure function analysis. Extrinsic dyes that label the nucleic acid alleviate those problems [3,7,8,15], but thus far, have failed to monitor complex structures such as the hammerhead ribozyme.

### SYBR Gold

To achieve kinetic measurements without substrate-labeling, we performed the band visualizations from post-migration staining of the gel with SYBR Gold. Imprecision in the measurement of the band intensity, whether systematic or random, would be directly reflected in a poor curve-fit of the data, while rates similar to those published from independent laboratories support the validity of the new band revealing methods [5, 12]. Nevertheless, we estimate that we can reliably quantify 1 pmol of ssDNA with SYBR Gold stain.

In order to test the validity of SYBR Gold gel staining as an alternative conventional gel-kinetic method to quantify nucleic acid kinetics, a simple DNA system was utilized as a working model. Typically, protein-less ribozyme systems are much easier to manipulate than the protein enzymes. Ribozymes are relatively easy to generate by *in vitro* transcription and can be renatured after ethanol precipitation, freezing or heating. The reduction of the sample complexity offered from a protein to RNA system is even greater with a DNA-only system. The general properties of the SYBR Gold quantification method had to be tested on a simple system where the activity rates were previously reported by a conventional radioactive assay.

### D-Zyme kinetics

In a conventional radioactive kinetic assay of cleavage, the longer radiolabeled substrate is separated from the shorter radiolabeled product, where the size of the non-labeled enzyme is irrelevant. The calculation of percent cleavage is based on the radioactive densitometry of the gel-separated substrate and product DNAs. To establish this technique as a valid DNA quantification method, a DNA-cleaving DNA enzyme molecule, D-Zyme, was used as the kinetic model [12]. The D-Zyme is a small, all-DNA sequence that has the simple structure of an asymmetric bulge around two long stems. Although the sequence and lengths of stems I and II could be highly diverse, the published sequence was used for direct comparison with the conventional radioactive kinetic technique [12]. The asymmetric bulge leads to a substrate of 37 nt-long, being slightly shorter than the enzyme of 40 nucleotides, the separation between the two was crucial in order to quantify the substrate without enzyme contamination. Although the proximity of the bands was close, the ImageJ quantification allowed isolation of individual bands. The kinetics were repeated several times and the average rate was 0.57 ± 0.15 min^-1^. This 25°C cleavage rate compares very well to the radioactivity published rates at 37°C and 45°C, 1.0 and 1.6 min^-1^ respectively that were determined with the use of radioactivity [12]. Fig 2C is representative of all gels and curve fitting, where most of the points are very close to the single-exponential curve-fit. To evaluate the precision and validity of the new staining method, the curve fitting standard deviation of each measurement was tabulated.

### 2-AP kinetics

To confirm the precision of this SYBR Gold quantification method, we also performed cleavage kinetics, where dA15 was substituted with the fluorescent base analog, 2-AP, at the cleavage site. The kinetic rate determined by the conventional discontinuous method was compared with that of the continuous method by use of a spectrofluorometer. As expected from the literature, changing the dA15 to 2-AP lowered the cleavage activity [12] to a cleavage rate of 0.21 ± 0.06 min^-1^, resulting in a 2.7-fold cleavage activity reduction.

The fluorescent base analog, 2-AP, is known to have its fluorescence quenched when intercalated, or stacked between other bases. The 2-AP at the cleavage site is flanked by the nucleotides dA16 downstream and dG14 upstream, which quench fluorescence when stacked. Upon catalysis, the 2-AP is unstacked in the product, leading to a fluorescence intensity increase, as shown in Fig 3A as well as reported in other 2-AP studies [16,17]. The real-time fluorescence kinetics was expected to have a similar rate of 0.2 min^-1^ but instead was much faster at 1.65 min^-1^.

This result is already under investigation. The initiation of the reaction by manual addition of zinc ions to the cuvette, rather than stopped-flow pumps prevent the recording of the initial 2-AP fluorescence intensities. It is however still possible to fit the data to a single-exponential function in Minitab in order to evaluate the cleavage rate. Stopped flow fluorescence spectroscopy would reduce the error in this measurement. Moreover, the DNA concentration dependency observed in Fig 4 is likely involved here as well as in the cuvette-spectrophotometer DNA samples, which were more diluted than the ones for the SYBR Gold gel staining kinetics.

### D-Zyme reaction conditions

Clearly, the SYBR Gold quantification method not only confirms the presence of nucleic acids cleavage activity, it also provides enough resolution to measure the rate kinetics with curve fitting. The cognate enzyme:substrate complex, D-zyme, has a catalytic activity (0.57 min^-1^ at 25°C), which is relatable to that of the one previously published (1.0 min^-1^ at 37°C) [12]. Although the radioactivity and the SYBR Gold methodology are very similar, they do not share the exact same reaction conditions in two major ways: temperature and DNA concentration. The known cleavage rates at 37°C (1.0 min^-1^) and 45°C (1.6 min^-1^) are considered relatively fast for nucleic acid catalysis and require short incubation period with many aliquots that would be taken almost immediately one after the other [12]. To slow-down the cleavage rate reaction without affecting its mode of action the incubation temperature was therefore lowered to 25°C. In the radioactivity experiment, most reactions contained 10 pmols of DNA in 30 μL of reaction (yielding a 0.3 μM DNA solution) that requires 1–2 mM of zinc for optimal activity in 2 minutes, while 0.5–5 mM zinc showed almost no activity for the same time period [12]. To be quantified by SYBR Gold, we estimated a minimum of 30 pmols of substrate per well was required, resulting to a reaction mixture of 3300 pmols of DNAs in 40 μL for a DNA concentration of 82.5 μM. While both reaction conditions were performed at the same pH of 7.05, there was minimum cleavage at 1 mM, 2 mM and 5 mM zinc chloride for the concentrated DNA reaction mixture. Small cleavage activity was observed at 10 mM, while the maximum activity was measured at 20 mM zinc chloride. This is a different outcome from the radioactive experiment, as suggested by the trend of zinc chloride saturation as function of total DNA in Fig 4 and the calculations below.

Assuming the D-Zyme activity requires a catalytic zinc, this increased dependence could be due to higher affinity for backbone-phosphate stabilization than the catalytic core. With the assumption that the zinc in this system acts both structurally by neutralizing the phosphate backbone charges, as well as being catalytic, it is possible that most of the catalytic zinc ions are chelated by the backbone phosphates and unavailable to catalyze the reaction. The overall negative charge of 77 nucleotide-DNA at 0.3 μM is 25 μM and a 1:1 charge ratio with zinc means that 1 mM zinc is in 80-fold excess over the DNA. The concentrated DNA of 82.5 μM represents the much larger 6.3 mM negative charges which would therefore require much more cations to stabilize its backbone. According to trends in Fig 4, such a hypothesis seem plausible as greater DNA concentration require more zinc chloride to achieve maximum activity. Additional magnesium or sodium ions did not reduce the amount of zinc required for optimum catalysis [12], suggesting that zinc ions have a greater affinity for this DNA than either sodium or magnesium ions. Little is known about nucleic acid catalysis and zinc, as there are very few reviews covering the deoxyribozymes involved with zinc [18]. Most biochemical catalysis involving zinc have been done with protein-based metalloenzymes [19–21]. Zinc has a filled *d* orbital (d10) and therefore does not participate in redox reactions but rather functions as a Lewis acid in accepting a pair of electrons [22]. It is an ion of borderline hardness and has high affinity for nitrogen and oxygen donor atoms as well as for sulfur as it has been found to bind to histidines, glutamates, aspartates and cysteines. When it is catalytic, it is exposed to the solvent and generally one water molecule completes the coordination [20].

### Hammerhead kinetics

With the aim of confirming SYBR Gold post-staining as a versatile quantification method, we compared the hammerhead ribozyme cleavage rates measured from our alternative method to that of standard radioactivity [13]. At 0.5 mM MgCl_2,_ this *trans*-acting hammerhead RzB has an observed rate of cleavage of 1.8 min^-1^ at 37°C. The cleavage rate of this fast-cleaving hammerhead ribozyme is unknown at 10 mM MgCl_2,_ where we measured a cleavage rate of 7.6 min^-1^ at 37°C. Such a fast cleavage rate confirms the versatility of the new labeling-free kinetic method for not only short DNAs, but now very short RNA fragments can have their intensities measured by SYBR Gold post-migration staining. It is remarkable that the short 18-nucleotide-long substrate as well as both 7-nt and 11-nt-long RNA products are detectable and quantifiable.

### Advantages

The advantages of this quantification method are numerous. The absence of substrate labeling is both fast and structurally worry-free. It is a method easily adapted from conventional radioactivity gel-kinetics without the use of expensive instrumentation to read radioactivity or fluorescent gels. A conventional transilluminator attached to a camera Gel Doc System is sufficient to acquire the image. SYBR Gold is much safer to use than radioisotopes and without regulations about storage, handling, decontamination and waste. It is a method that can provide rapid confirmation of catalysis and it offers the precision required to perform curve-fitting of the data to measure rates. Moreover, it is a method that works with simple DNA structures as well as complex RNA structures such as the hammerhead ribozyme. It is a technique truly accessible to any lab currently doing gel kinetics, with minimal instrumentation required.

### Drawbacks and improvements

Even though this new method requires a much larger amount of nucleic acid to measure the kinetic by comparison with radioactive or fluorescent-labeled substrates, it can however, as it is the case in this study, reveal new details that are concentration-dependent. The large amount of DNA required when compared with the 32-P radioactivity experiment rather illustrates the extremely high sensitivity offered by use of radioisotopes. Following radioactive labeling, fluorescence generally offers the next best signal-to-noise ratio. Until the development of more efficient fluorophore dyes, SYBR Gold is an excellent choice to quantify bands for nucleic acid kinetics. According to our results, the analysis could be simplified with greater separation between the enzyme and the substrate (S1 Fig), which can be performed with different gel sizes, acrylamide composition, or DNA lengths.

It is also imperative to perform the kinetics with gel-purified samples, as well as ensuring a sufficient separation between the sizes of the enzyme and the substrate. While performing the gel purifications by UV Shadowing, the band seemed unique without synthesis failure. We therefore attempted the kinetics without gel purification, where the sensitivity of SYBR Gold identified all synthesis-failure of shorter DNA lengths, that were previously undetected by UV shadowing. The image of the gel showed a ladder at every single nucleotide with accumulations at the product, substrate and enzymes rendering the analysis impossible.

We demonstrated the use of SYBR Gold post-migration staining to quantify RNA and DNA bands for cleavage kinetics analysis without substrate labeling. Although this method is comparatively less sensitive than radioactivity or fluorescence labeling, it has several advantages such as: i) ease of use; ii) cost effectiveness; iii) no half-life decay; iv) absence of substrate labeling; v) precision enabling curve fitting; vi) versatility with non-structured and structured RNAs or DNAs.

## Supporting information

S1 Fig Kinetics of the DNA Enzyme with a shorter substrate (S -4). (TIF)

S2 Fig Kinetics of the DNA Enzyme from 2-AP fluorescence intensity unquenching. (TIF)

S3 Fig Kinetics of the 2-AP substrate annealing and cleavage reaction. (TIF)

S1 File (PDF)
