# Supplementary figures and images for: A simple and affordable kinetic assay of nucleic acids with SYBR Gold gel staining
Source: PLoS One. 2020 Mar 3;15(3):e0229527. doi: 10.1371/journal.pone.0229527 (PMC7053750; doi:10.1371/journal.pone.0229527)

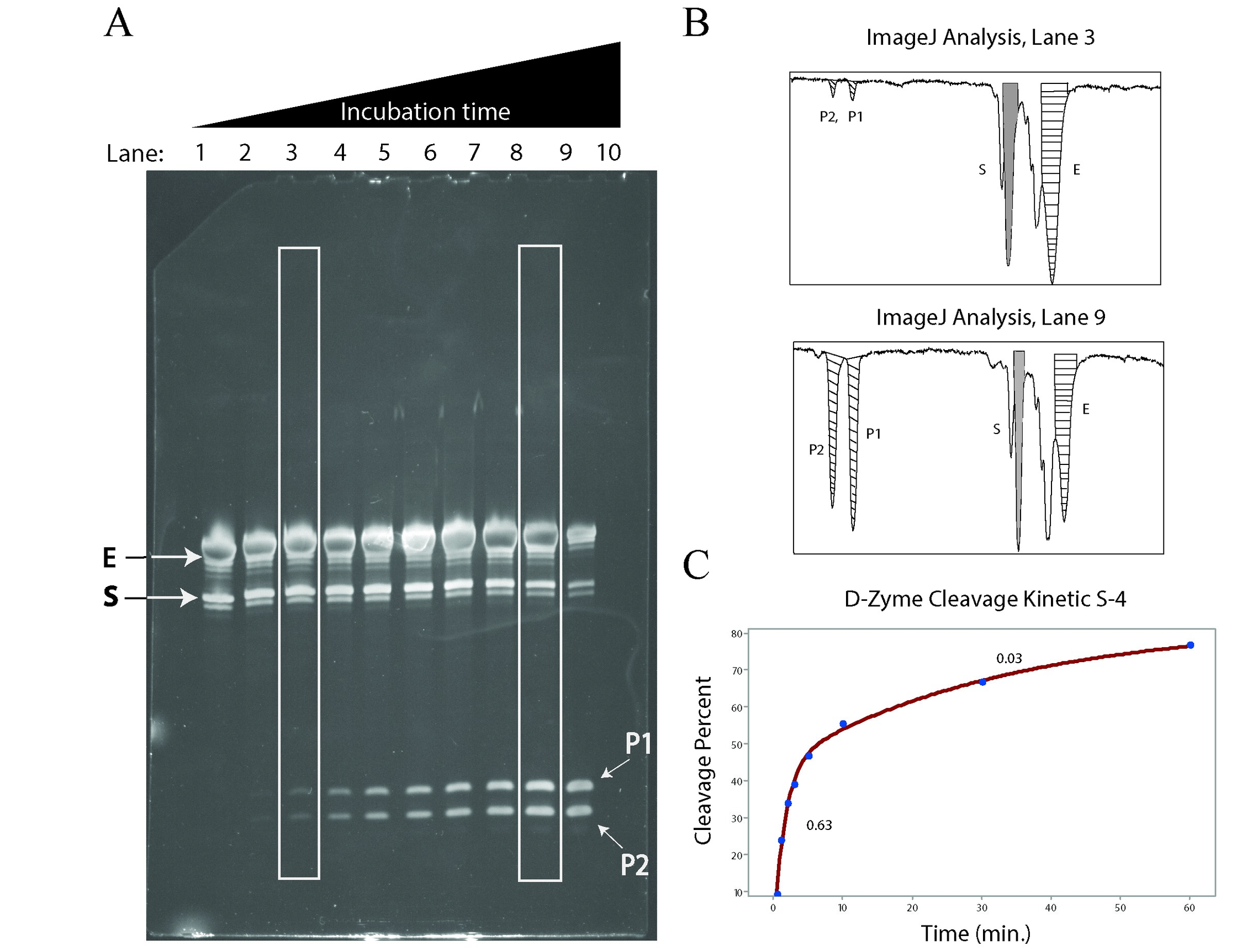

Supplement: S1 Fig — (TIF) [file pone.0229527.s001.tif]

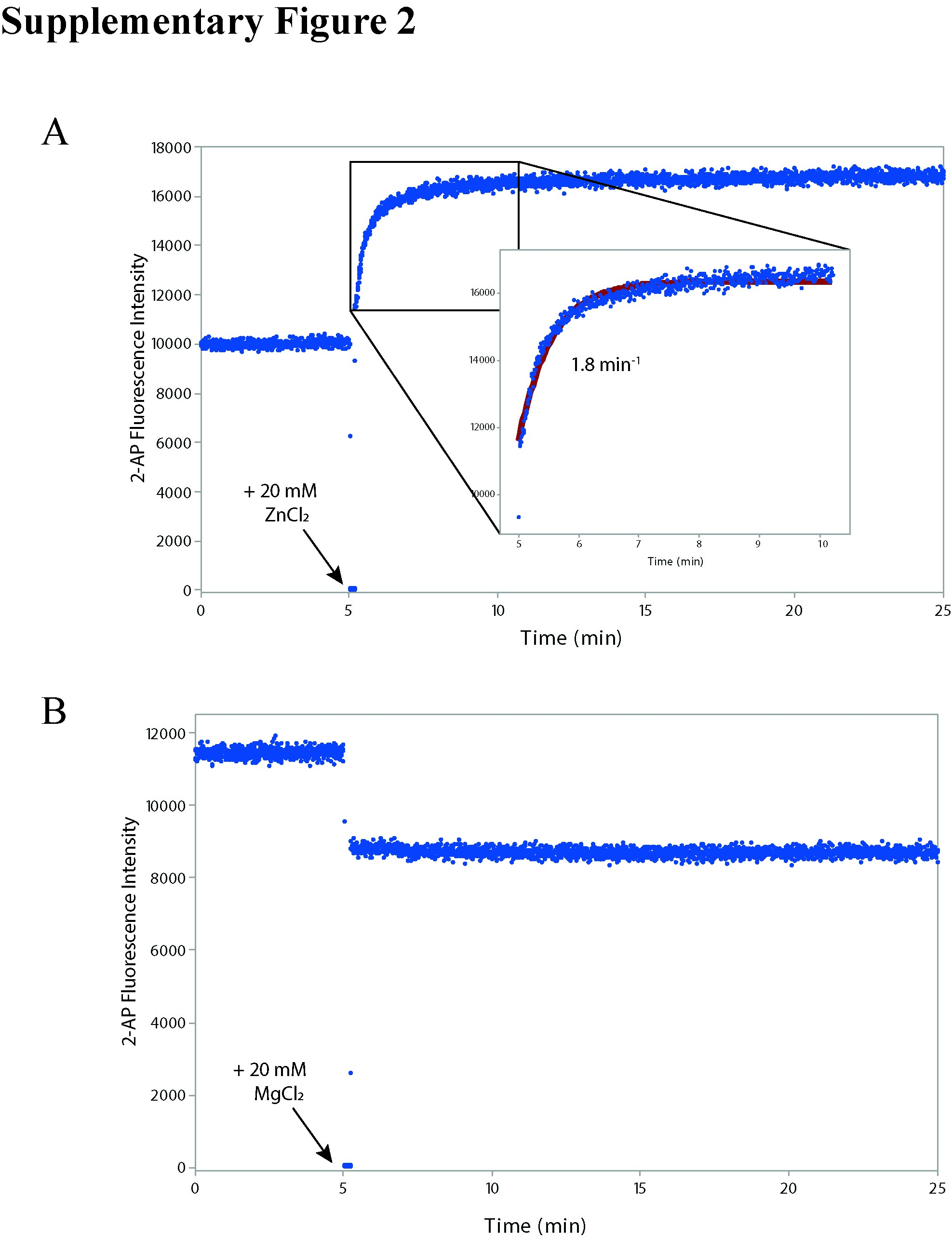

Supplement: S2 Fig — (TIF) [file pone.0229527.s002.tif]

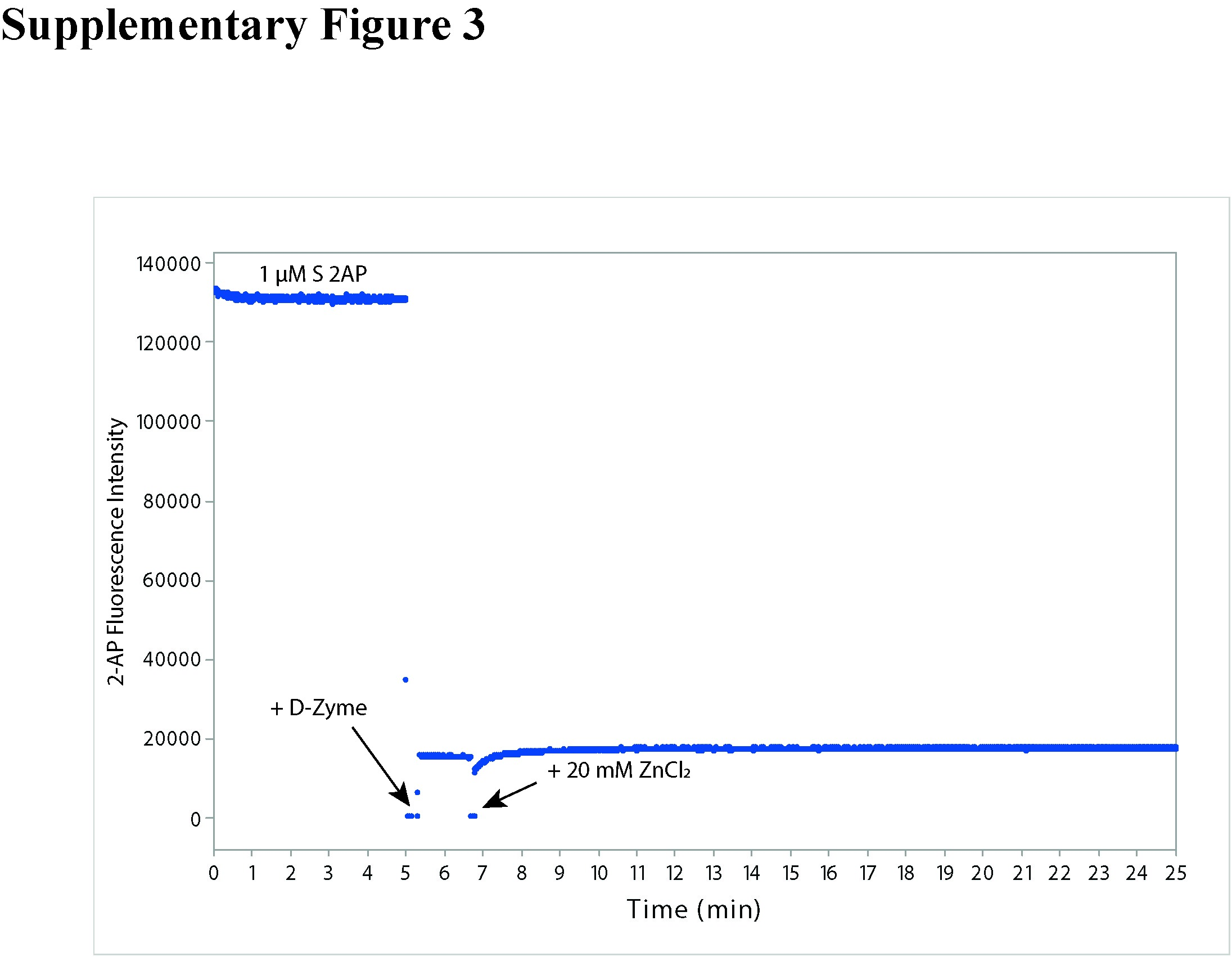

Supplement: S3 Fig — (TIF) [file pone.0229527.s003.tif]
