# Supplementary material for: A simple and affordable kinetic assay of nucleic acids with SYBR Gold gel staining
Source: PLoS One. 2020 Mar 3;15(3):e0229527. doi: 10.1371/journal.pone.0229527 (PMC7053750; doi:10.1371/journal.pone.0229527)
Supplement: S1 File — (PDF) [file pone.0229527.s004.pdf]

Supplementary Information

DNA Purification

DNA oligonucleotides were purchased from Integrated DNA Technologies (Coralville, Iowa) and purified on 10% 8 M urea polyacrylamide gel electrophoresis (PAGE) 19:1 acrylamide:bisacryamide in TBE buffer (89 mM Tris, 89 mM Boric Acid and 2 mM EDTA) for 1.5 hours at 20W. Visualization of the DNA by UV Shadowing was performed by placing the gel on a TLC plate, containing the fluorescent dye F254, and illuminated by a 6W 254 nm lamp epi-illumination. The DNA of appropriate size were cut out of the gel with an autoclaved razor blade.

Electroelution

Samples were placed into the wells of an IBI electroeluter [1], and electroeluted for 45 minutes at 180V in diluted TBE buffer (22 mM Tris, 22 mM Boric Acid and 0.5 mM EDTA). To trap the eluting DNA, chilled 8M NH_4OAc was inserted into the V-tubes to prevent migration. Tips were used to block the V-tubes and TBE was removed until below the well levels. The DNA/salt was pipetted-out with gel-loading tips and transferred into a microcentrifuge tube for ethanol precipitation.

Crush and soak

Alternatively to electroelution, DNA was recovered from the gel by passive diffusion in tumbling for 3 hours at room temperature with an elution solution of 0.3M NaCl, 0.001% SDS and 0.1mM EDTA followed by ethanol precipitation. The samples were resuspended in nuclease-free water. Absorbance values were determined at 260 nm with a spectrophotometer.

Densitometry

The open source software, ImageJ (version 1.52K), was utilized to quantify the brightness of the bands. After converting the tiff image to 32-bit, a rectangular selection was made around the lane of

interest (Fig 2A), including DNA of all sizes and background. The analysis of the rectangle produces a graph of the different intensities as a function of distance, separated by the background. The area under the curves corresponding to the bands of interests were sectioned off using the line tool. The area is calculated by using the wand tool for each sectioned-off band (Fig 2B). The numbers produced by ImageJ were exported to a Minitab spreadsheet, to calculate cleavage percentage. This process was repeated for each lane.

ImageJ is a freely available open source software, developed in Java, and hosted by the NIH, USA. The advantages of the software are its high customizability and relative ease of use. Unfortunately, sometimes lane identification fails, or produces duplicate analyses. This is easily fixed by reselecting and processing only one lane at a time. Unlike PhosphorImager, the user must manually set the background level by drawing a horizontal line. Additionally, two vertical lines must be drawn to separate the substrate from other DNAs, by extrapolation up to the background line (Fig 2B). This extrapolation is imperative to find the area under the curve, strictly for the band of interest.

Quality of fit

As it is the case for most discontinuous kinetic assays, the quality of curve fitting can be improved by increasing the number of data points. As exemplified in Fig 2C, not all the data points are fit with great precision. Those variations are quantified with curve fitting error provided by Minitab where the error percentage was mostly below 20%. For example, we excluded the data from a kinetic run that had too few points for the fit and yielded a curve fitting error greater than 25%.

During the ImageJ analysis, we encountered two major concerns that affected the quality of the data. First, was the substrate of 37 nucleotides, which is very close to the large ten-fold excess enzyme of 40 nucleotides. The large excess of enzyme sometimes co-migrated with the substrate, invalidating the data. To manage and circumvent this size similarities, we did a kinetic with a substrate four nucleotides shorter where two base pairs were missing on each end. The 40 nucleotides enzyme now migrates much farther from the 33 nt nucleotides substrate and significantly improves the analysis, S1 Fig. Second was the need to set the appropriate limits on the width of the substrate spot. As seen in Fig 2B, vertical lines

are extrapolated up to the background level to “close-off” the area under the curve. The width of the area from the products provides a good estimation for determination of the width of the substrate peak and its size can easily be overestimated leading to a smaller cleavage fraction.

Those two factors are the main contributors for the curve fitting errors we observed. Still, it should be noted that regardless of those errors, the trend of the cleavage reaction is clearly following a single-exponential curve fit, a trend that would not be expected to happen for a method with high variations in measurements.

2-AP fluorescence

In order to ensure that the fluorescence kinetic rate observed indicates the cleavage of the substrate that translate into a product that prevents the quenching of the 2-AP, critical controls were provided in S2 and S3 Figs.

The addition of zinc chloride initiates the substrate cleavage while magnesium chloride is known to not contribute to the reaction. Therefore, a 25-minutes kinetic run with the addition of either zinc chloride or magnesium chloride after 5-minutes of equilibration was performed in S2 Fig. The single-exponential curve-fitting strictly for the zinc chloride provides a strong evidence that the cleavage of the substrate leads to the un-quenching of the 2-AP. Moreover, S3 Fig indicates that the free substrate is almost 10-times more fluorescent than when it becomes base-paired with the D-Zyme at 5 minutes. Once again, the addition of zinc chloride at around 7 minutes shows a rapid curved progression of the reaction.

SUPPLEMENTARY FIGURES CAPTIONS

S1 Fig. Kinetics of the I-R3 D-Zyme with a shorter substrate. A. The 4-nucleotides shorter substrate (S -4). Incubation time increases with increasing wells, from 0 sec in well #1 and 60 min in well #10. The enzyme strand is shown at 40 nt length followed by the substrate strand at 33 nt, and the two products at 17 and 16 nt. Selected wells highlight the significant difference in band intensity between two time points,

correlating to an increased amount of product as the reaction progressed. More importantly is the greater separation between the enzyme and the substrate as compared to Fig 2. **B**. The ImageJ graphs corresponding to the lanes highlighted show a greater distinction between the enzyme and substrate peaks. Hashed segments represent the product, gray represents the substrate, and striped represents the enzyme. **C**. Double exponential curve-fitting using Minitab of densitometry analysis for D-Zyme kinetics with a fast rate of 0.63 min^{-1} and a slow rate of 0.03 min^{-1}.

S2 Fig. **Kinetics of the I-R3 D-Zyme from 2-AP fluorescence intensity unquenching. A.** After 5 minutes of equilibration of the enzyme annealed to the substrate in 50 mM HEPES, 100 mM NaCl, pH 7.05 at 25ºC, the spectrofluorometer lid is open (indicated by an arrow at 5 minutes) while still acquiring, resulting in datapoints at 0 fluorescence intensity for a few seconds, followed by the addition of zinc chloride to a final concentration of 20 mM. The increase of fluorescence intensity is fitted to a single exponential function in Minitab for a rate of 1.8 min^{-1} as shown in the zoomed inset. **B.** After 5 minutes at 25ºC, the spectrofluorometer lid is open (indicated by an arrow at 5 minutes) while still acquiring, resulting in datapoints at 0 fluorescence intensity for a few seconds, followed by the addition of magnesium chloride to a final concentration of 20 mM. The variation of 2-AP fluorescence intensity is due to dilution rather than quenching.

S3 Fig. **Kinetics of the 2-AP substrate annealing and cleavage reaction**. The initial 2-AP fluorescence is that of the free 1 μM 2-AP substrate in 50 mM HEPES, 100 mM NaCl, pH 7.05 at 25ºC. At 5 minutes, an arrow indicates opening of the lid followed by the addition of 10 μM Enzyme, which quenches the 2-AP fluorescence drastically. A few minutes later, zinc chloride is added to initiate the cleavage reaction, which shows an exponential increase of fluorescence as reported in S2A Fig.

References

1. Zarzosa-Álvarez AL, Sandoval-Cabrera A, Torres-Huerta AL, Ma. Bermudez-Cruz R. Electroeluting DNA Fragments. J Vis Exp. 2010; e2136. doi:10.3791/2136

S1 Fig

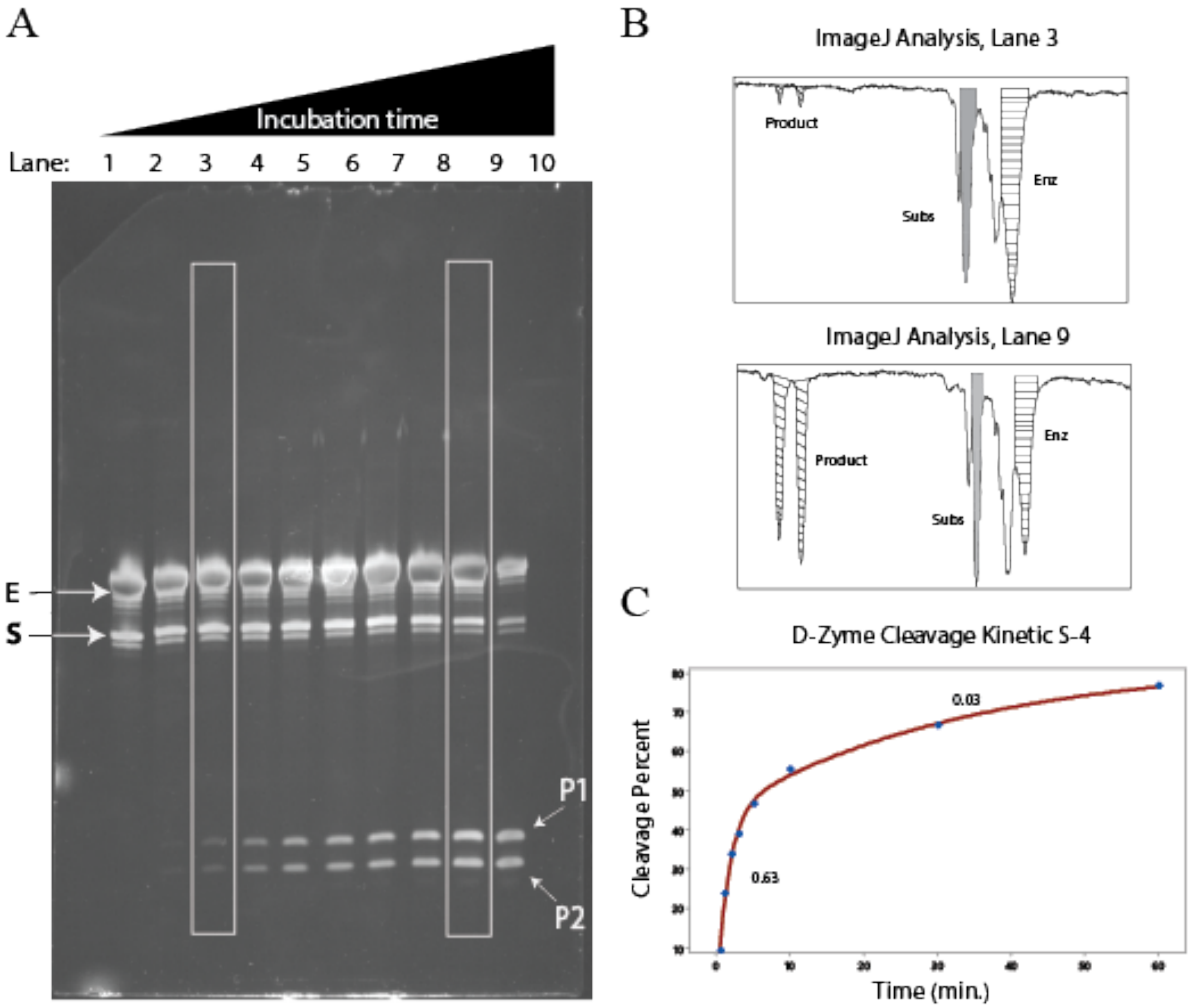
A
Incubation time
Lane: 1 2 3 4 5 6 7 8 9 10
E
S
P1
P2
B
ImageJ Analysis, Lane 3
Product
Subs
Enz
ImageJ Analysis, Lane 9
Product
Subs
Enz
C
D-Zyme Cleavage Kinetic S-4
0.03
0.63
Cleavage Percent
Time (min.)

S2 Fig

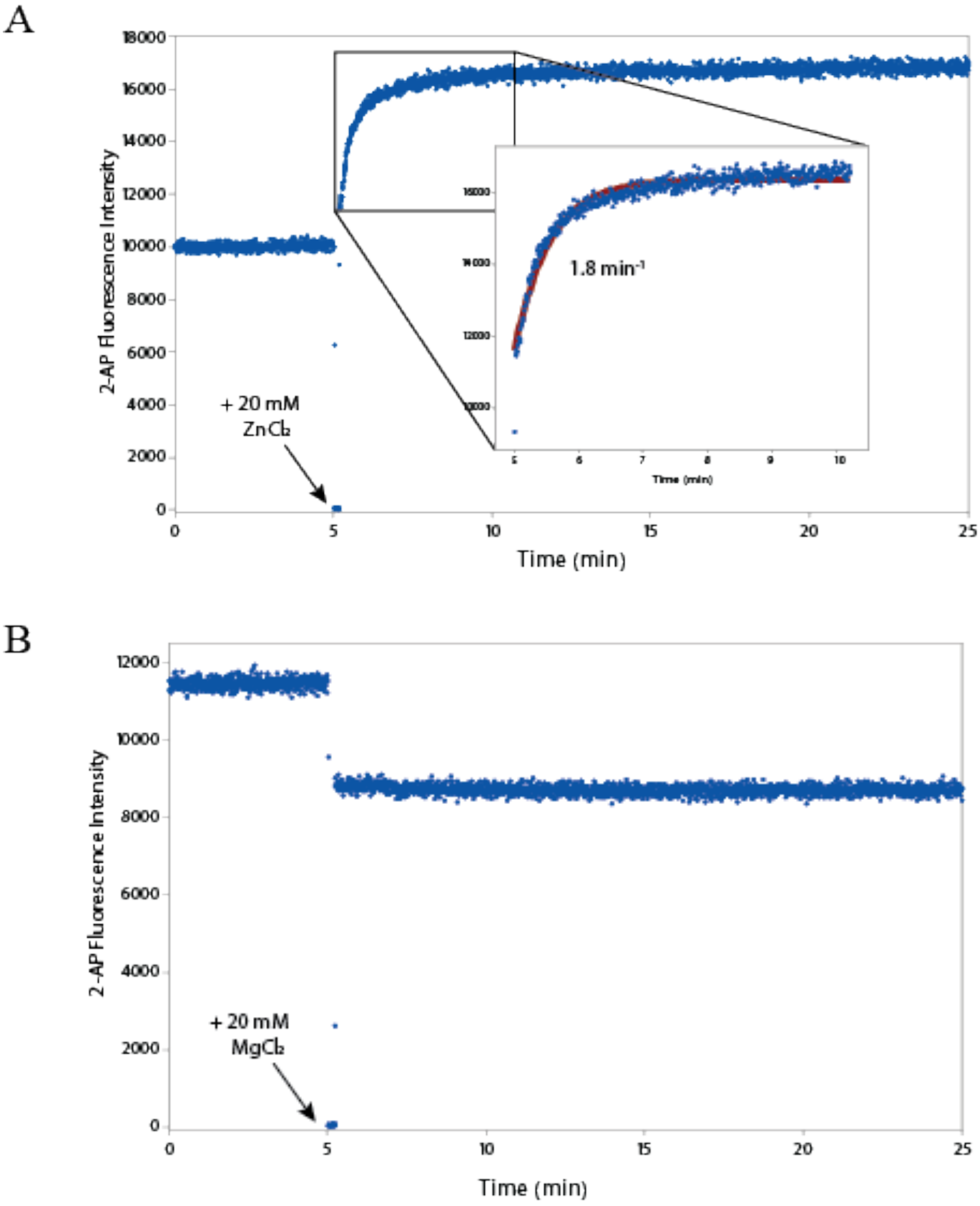

A
2-AP Fluorescence Intensity
Time (min)
+ 20 mM ZnCl2
1.8 min-1
B
2-AP Fluorescence Intensity
Time (min)
+ 20 mM MgCl2

S3 Fig

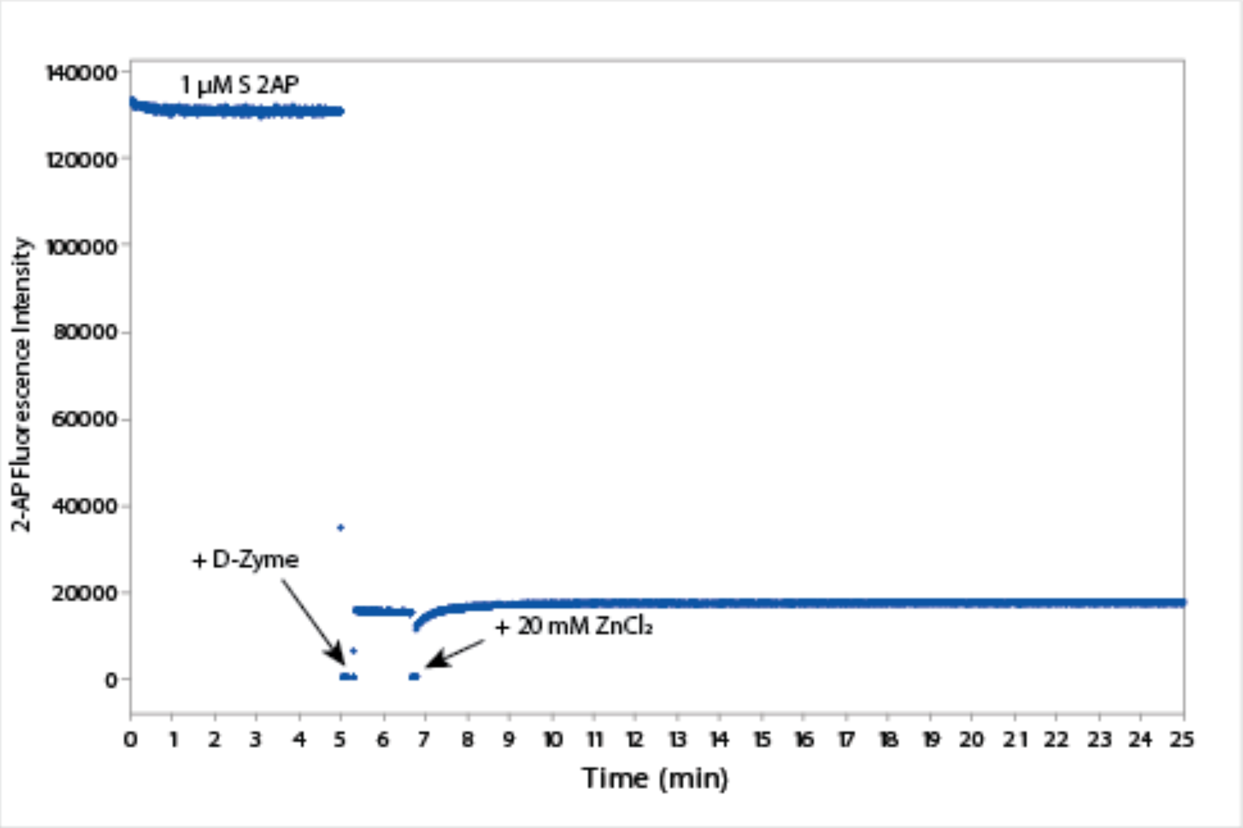
1 µM S 2AP
+ D-Zyme
+ 20 mM ZnCl₂
2-AP Fluorescence Intensity
Time (min)
140000
120000
100000
80000
60000
40000
20000
0
0 1 2 3 4 5 6 7 8 9 10 11 12 13 14 15 16 17 18 19 20 21 22 23 24 25
